# Risk Factors and Role of Antibiotic Prophylaxis for Wound Infections after Percutaneous Endoscopic Gastrostomy

**DOI:** 10.3390/jcm12093175

**Published:** 2023-04-28

**Authors:** Antonia Mondorf, Clara Amini, Christiana Graf, Florian Alexander Michael, Irina Blumenstein, Michael Jung, Mireen Friedrich-Rust, Daniel Hack, Silke M. Besier, Michael Hogardt, Volkhard A. J. Kempf, Stefan Zeuzem, Christoph Welsch, Jörg Bojunga

**Affiliations:** 1 Department of Internal Medicine 1, Goethe University Hospital Frankfurt, Goethe-University, 60596 Frankfurt am Main, Germany; 2 Institute for Medical Microbiology and Infection Control, University Hospital Frankfurt, Goethe-University, 60596 Frankfurt am Main, Germany; 3 University Center of Competence for Infection Control of the State of Hesse, 60596 Frankfurt am Main, Germany

**Keywords:** percutaneous endoscopic gastrostomy (PEG), wound infection, head and neck cancer, radio-chemotherapy, antibiotic prophylaxis

## Abstract

Background and study aim: The incidence of wound infections after percutaneous endoscopic gastrostomy (PEG) varies widely in recent studies. The present study systematically investigates the underlying risk factors for the development of wound infections in a large cohort of patients over a long-term follow-up period. Patients and Methods: A retrospective cohort study of patients undergoing PEG insertion using either the pull or push technique was conducted and patients followed up for 3 years. Tube-related wound infections were identified, and pathogens regularly cultured from wound swabs. Adjusted analysis was performed via univariate and multivariate logistic regression analysis. Results: 616 patients were included in this study. A total of 25% percent of patients developed wound infections upon PEG tube insertion and 6.5% showed recurrent infections. Nicotine abuse (*p* = 0.01), previous ischemic stroke (*p* = 0.01) and head and neck cancer (*p* < 0.001) showed an increased risk for wound infection after PEG placement. Moreover, radio-chemotherapy was associated with the occurrence of wound infections (*p* < 0.001). Infection rates were similar between pull and push cohorts. The most common bacterial pathogen detected was Enterobacterales (19.2%). *Staphylococcus aureus*, *Pseudomonas aeruginosa* and *enterococci* were frequently detected in recurrent infection (14.2%, 11.4% and 9.6%, respectively). Antibiotic prophylaxis showed no effect on infection rates. Conclusions: Wound infections after PEG placement are common and occasionally occur as recurrent infections. There is potential for improvement in everyday clinical practice, particularly regarding antibiotic prophylaxis in accordance with guidelines.

## 1. Introduction

Since its introduction in 1980, percutaneous endoscopic gastrostomy (PEG) has proven to be a highly effective method for long-term enteral nutrition [1]. Indications for PEG insertion are usually in patients who are unable to take oral nutrition to adequately meet their caloric and nutritional needs [2,3]. The most common underlying diseases in this context are dysphagia due to a neurological or traumatological cause and cancer patients, in particular those patients with head and neck cancers (HNC). In the latter, a PEG is performed before the start of radio-chemotherapy to ensure continuous oral nutrition and prevent greater weight loss in the context of the underlying disease and the tumor therapy [4,5].

The PEG can be placed either via the “pull technique”, the most common technique, or, in the case of stenoses in the esophagus and oral cavity, through direct puncture (“push technique”) after prior gastropexy. Both techniques have been shown to be safe within their respective indications [6]. Specific complications of PEG tube placement, such as the buried bumper syndrome (PEG internal bumper migrated and ingrown along the stoma tract), gastric ulcers, or dislocation of the tube, are rare overall [7,8]. The most common and clinically relevant complications that occur post-intervention are wound infections [9,10]. According to the current literature, the incidence of wound infections after PEG placement varies widely, ranging from 4% to 30% [11]. The high variability of infection rates in different studies is probably due to different patient cohorts and different measures for infection control. The latter in particular are often inconsistent and are probably too rarely implemented in accordance with guidelines as well as the local bacterial resistance situation [12]. The currently known risk factors for wound infections include diabetes mellitus, malignancies and immunosuppressive therapies [9,13]. For example, an increased risk for wound infections was observed in patients with HNC receiving simultaneous radio- and chemotherapy [14]. However, there are few large studies that systematically address risk factors for wound infections after PEG placement. A 2013 Cochrane review of 11 studies and 2 meta-analyses showed a benefit of peri-interventional antibiotic prophylaxis with respect to the primary endpoint of post-interventional wound infections, within the first 30 days after PEG placement [11]. Based on this Cochrane analysis, international guidelines recommend prophylaxis with a penicillin-based or a cephalosporin-based therapy 30 min before PEG placement [2,12,15]. This excludes patients with pre-existing antibiotic therapy, who do not need separate prophylaxis. Despite some studies and evidence for the benefit of prophylactic antibiotic administration before PEG placement, there are only few microbiological data on the pathogen spectrum in wound infections after PEG placement and, thus, on antibiotic prophylaxis that may need to be optimized. A study from the United States showed a high proportion of staphylococci in wound infections after PEG placement [16], while studies from India and Korea mainly detected *Pseudomonas aeruginosa* and *Klebsiella* spp. [17,18]. Some studies showed a high proportion of yeast infections, mainly with *Candida* spp. [19,20]. However, it remains unclear to what extent the detection of fungal infections contributes in a clinically relevant way to the observed wound infections and if antifungal therapy is required.

The aim of this retrospective study was to characterize risk factors for the development of wound infections and the benefit of antibiotic prophylaxis in a large cohort of 616 patients after PEG placement. In particular, the pathogen spectrum in wound infections within 7 days after PEG placement was characterized and compared with the risk factors identified here and the respective anti-infective prophylaxis used.

## 2. Patients and Methods

### 2.1. Study Population

All patients 18 years of age or older who had a percutaneous endoscopic gastrostomy (PEG) tube inserted at Frankfurt University Hospital between 1 January 2014 and 31 December 2017, were included in this retrospective, single-center study with a follow-up observation period of three years. Before the start of the study, approval was obtained from the local ethics committee of the Frankfurt University Hospital (HIC approval no. 76/18).

### 2.2. PEG Placement and Recording of Infectious Complications

PEG tube placement was performed according to standard operating procedures by the endoscopy department using commercially available PEG tubes (Freka PEG CH 15, Fresenius-Kabi, Bad Homburg, Germany). PEG placement was performed by two experienced physicians. Two standard PEG insertion techniques were used, the “pull” technique (gastroscope-assisted) and the “push” technique (fluoroscopy-assisted direct puncture technique). The “push” technique was used in patients in whom the “pull” technique was not possible due to mechanical obstruction such as stenosis or tumor. Prior to PEG placement, local disinfection of the puncture site was performed according to common hygiene standards (triple mechanical disinfection with sterile compresses and an alcohol-based, dyed disinfectant solution). Prior to PEG insertion, the upper gastrointestinal tract was examined by esophagogastroscopy. The stomach was inflated with air and a suitable puncture site was identified using diaphanoscopy. If the “pull” technique was used, after local anesthesia and puncture incision, a puncture cannula was advanced into the stomach under endoscopic control and the puncture needle was removed from the cannula. A thread was inserted via the cannula, grasped with the endoscope and passed out through the esophagus and mouth. The thread was then attached to the feeding tube with an internal fixation plate. By pulling the distal end of the thread, the feeding tube was passed out over the stomach wall and secured using an internal fixation plate and shortened to a suitable length. In the “push” technique, a gastropexy was performed before the PEG insertion. This involved using a gastropexy device to secure the stomach wall to the abdominal wall. The feeding tube was then placed directly through the stomach wall using a puncture trocar, at the same time as the gastropexy sutures were pulled upwards. The feeding tube was fixed with a balloon catheter, which was blocked by the trocar after insertion. After PEG insertion, patients were trained regarding PEG handling, and followed up by our nutritional medicine outpatient clinic. All patients were followed at least once approximately 14 days after PEG placement and if complications occurred. The occurrence of wound infections at the PEG insertion site was determined by nursing staff specially trained in wound management, and the severity of the wound infection was categorized in 3 severity levels: Grade 1: local peristomal infection with pain, local redness, and hyperthermia; Grade 2: presence of pus, and fever with need for systemic antibiotic therapy; and Grade 3: additional meeting of sepsis criteria (according to Sequential Organ Failure Assessment (SOFA) Score [21]).

### 2.3. Clinical Microbiology Procedures

In case of wound infections, swabs were taken from the PEG insertion site and were processed in the Institute for Medical Microbiology and Infection Control as part of routine microbiological diagnostics. Species identification was conducted using matrix-assisted-laser desorption ionization–time of flight analysis (MALDI–TOF) and VITEK2 (bioMérieux, Nürtingen, Germany). Antibiotic susceptibility testing was performed according to Clinical Laboratory Standards Institute (CLSI) guidelines using VITEK2 and antibiotic gradient tests (bioMérieux) [22]. Lateral flow assays (Hardy, Santa Maria, CA, USA) were used to detect the following carbapenemases: NDM, KPC, OXA–48, VIM and IMP. Carbapenemase-encoding genes were detected via PCR analysis and subsequent sequencing from carbapenem-resistant *Enterobacterales* including the *bla* genes for carbapenemases NDM, VIM, IMP, OXA–48-like, and KPC as well as OXA–23, OXA–24, and OXA–58 for *A. baumannii.* All laboratory testing was performed under strict quality control criteria (laboratory accreditation according to ISO 15189:2011 standards [23]) at the Institute for Medical Microbiology and Infection Control, University Hospital Frankfurt am Main, Germany.

A bacterial isolate was considered to be a multidrug-resistant organism (MDRO) if it had an acquired non-susceptibility to at least one in three or more antimicrobial categories: extended-spectrum beta-lactamase (ESBL) producing *Escherichia coli* and *Klebsiella pneumoniae*, carbapenem-resistant *Acinetobacter baumanii* and *Pseudomonas aeruginosa* as well as *Stenotrophomonas maltophilia*, *Achromobacter xylosoxidans*, and de-repressed chromosomic AmpC ß-lactamase-producing *Enterobacterales* (*Enterobacter* spp.), vancomycin-resistant *Enterococcus faecium* (VRE) and methicillin-resistant *Staphylococcus aureus* (MRSA).

Screening for MDRO was performed according to the national recommendations of the Organization for Hospital Hygiene and Infection Prevention (KRINKO) [24]. Accordingly, patients with already-known MDRO colonization, patients with a hospital stay longer than 3 days in the last 12 months, patients before admission to an intensive care unit, patients with chronic care needs, and patients with contact to the health care system of countries with high MDRO prevalence were screened. Swabs were taken from the nose (for MRSA only), throat, tracheal secretions, and wounds, and a skin swab (axillae/groin/perianal) and deep rectal swab were also taken.

### 2.4. Statistical Analysis

Patient data were collected using the ORBIS electronic medical record (Afga Healthcare, Düsseldorf, Germany). Statistical analyses were performed using Microsoft Excel (version 2206 Microsoft, Redmond, Washington, DC, USA) and BiAS (version 11.12, Frankfurt am Main, Germany). Continuous data are presented as mean ± standard deviation and median with minimum and maximum depending on the presence of a standard normal distribution. Categorical data are presented as frequencies and percentages. In the absence of a normal distribution, continuous data were analyzed using the Wilcoxon-Mann–Whitney U. Associations between categorical data were determined with the chi-square (χ^2^) test. For categorical variables with an expected frequency of <5, Fisher’s exact test was applied. Univariate and multivariate logistic regression analyses were performed. All tests were two-sided and performed at a 5% significance level.

## 3. Results

This study included a total of 616 patients who underwent PEG tube insertion between 1 January 2014 and 31 December 2017. The follow-up period in this study for each patient was three years. Baseline patient characteristics are shown in Table 1. The median age of patients was 66 years with a total of 68.5% (*n* = 422) male patients. In 59.1% (*n* = 364) of patients, there was an indication for PEG placement due to planned radio-chemotherapy of tumors in the head and neck region.

224 wound infections were registered after PEG placement in 154 of 616 patients (25%). During follow-up, recurrent infections after PEG insertion occurred in 40 patients (6%). The overall complication rates are summarized in Table 2.

Patients with head and neck cancer show high risk for wound infections after PEG placement. In univariate analysis, patients with nicotine abuse (*p* = 0.01), previous ischemic stroke (*p* = 0.01) and HNC (*p* < 0.001) showed a significantly increased risk for wound infection. Multivariate regression analysis identified only HNC as a risk factor for wound infection after PEG placement (Table 3). In contrast, other malignancies, in particular esophageal cancer, did not show increased infection rates after PEG placement. A subgroup analysis in HNC patients identified additional risk constellations in this patient group (Supplementary Table S1). According to this subgroup analysis, wound infections after PEG placement seem to occur more frequently in HNC patients with additional risk factors being liver cirrhosis or Parkinson’s disease (*p* = 0.03 and *p* = 0.04, respectively).

Moreover, our data showed a statistically significant association between the occurrence of wound infections after PEG placement and radio- and chemotherapy (33.5%, *p* < 0.001), but not with radiotherapy alone (22.2%, *p* = 0.45) or surgery (18.8%, *p* = 0.22) (Table 4). Importantly, we could not find any correlation between the occurrence of wound infections and the technique of PEG placement (*p* = 0.85), pull technique (*n* = 561, 91.0%) versus push technique (*n* = 52, 8.4%). In three patients, it was not possible to retrospectively clarify which technique was used for PEG placement. These patients were excluded from our analysis. The subgroup of HNC patients showed similar results, pull technique (*n* = 326, 90.3%) versus push technique (*n* = 35, 9.6%) (*p* = 0.97).

Spectrum of detected bacteria: Pathogens/Bacteria were systematically isolated from wound swabs of the 224 wound infections confirmed in 154 patients after PEG placement. We detected 281 bacterial species and 121 fungal species (refers to the detection of individual pathogens in the case of mixed infections). The most common bacterial pathogens detected in wound swabs were *Enterobacterales* (*n* = 54, 19.2%), followed by bacteria of the normal skin flora (*n* = 51, 18.2%). Compared to patients without head and neck cancer, HNC patients showed a tendency for enterobacteria to be more frequently detected, as well as *S. aureus* and *viridans streptococci* (*p* = 0.036). In contrast, *enterococci* and *Pseudomonas aeruginosa* were detected more frequently in patients without HNC (Table 5). *Candida* spp. (*Candida albicans* 60%, *Candida glabrata* 14.3%, *Candida krusei* 9.5%, *Candida tropicales* 2.4%, and other *Candida* spp. 14.3%) were detected frequently in 30.1%, with no significant difference in frequency demonstrated in patients with and without HNC, respectively (Table 5). The proportion of recurrent infections was significantly higher in HNC patients than in non-HNC patients (*n* = 36, 29.0% versus *n* = 4, 13.3%, *p* = 0.08). In recurrent wound infections, *S. aureus*, *Pseudomonas aeruginosa* and *Enterobacterales* were frequently detected (16.1%, 13.1% and 21.2%, respectively) We did not observe an increase in multidrug-resistant organisms (MDRO) in patients receiving antibiotic prophylaxis during PEG placement (Supplementary Table S2). Only a small proportion of MDROs were found in the PEG swabs in patients with pre-known MDRO colonization. Gram-negative pathogens with multidrug resistance (MRGN) were detected in two patients. One patient was infected with *Pseudomonas aeruginosa* resistant to piperacillin derivates, cephalosporin with extended spectrum and fluoroquinolones (3MRGN) in the wound swab, the second patient was infected with a carbapenem-resistant *Pseudomonas aeruginosa* (4MRGN). In both patients, the corresponding MRGN had previously been detectable in the throat swab and upper respiratory tract, respectively. Noteworthily, HNC patients had significantly fewer MDROs (*p* < 0.001), except for the number of methicillin-resistant *S. aureus* (MRSA) colonizations (*p* = 0.55).

Peri-interventional antibiotics did not show a reduction in wound infections after PEG placement. In our cohort, only 52.3% of patients (*n* = 248) received guideline-based anti-infective prophylaxis during PEG placement. In addition to third-generation cephalosporins recommended in the guidelines, carbapenems (*n* = 64, 13.5%), fluoroquinolones (*n* = 57, 10.9%), and penicillin derivatives (*n* = 52, 10.9%) were used, mostly as a continuation of anti-infective therapy for other pre-existing infections. To analyze the immediate impact of prophylactic antibiotic administration in the context of PEG tube placement, the incidence of infections at the PEG entry site within the first 7 days after intervention was studied. We did not observe a significant reduction in wound infection rates in patients with antibiotic prophylaxis as compared to patients without antibiotic prophylaxis (*p* = 0.31). However, the number of severe infections (defined by the indication for systemic antibiotic therapy) seems to be reduced in patients with antibiotic prophylaxis compared to patients without antibiotic prophylaxis (7.1%, *n* = 34 and 11.3%, *n* = 16, respectively, *p* = 0.1) (Table 6). In the subgroup analysis of HNC patients, 16.5% of patients (*n* = 60) developed wound infection after 7 days. Again, antibiotic prophylaxis did not show any effect on infection rates (297 patients with prophylaxis, 16.2% (*n* = 48) infections versus 67 patients without prophylaxis, 17.9% (*n* = 12) infections) (*p* = 0.73).

To further evaluate the effect of guideline-based antibiotic prophylaxis, we performed a subgroup analysis of the corresponding patient population from our study. In patients who received a single course of antibiotics with ceftriaxone, according to guideline recommendations (*n* = 248 patients, 52.3%), wound infections did not occur significantly less frequently compared to patients who were treated outside the guidelines (Table 7). In the subgroup analysis of HNC patients, those receiving guideline-adherent prophylaxis had a lower rate of infection (14.4%, *n* = 29 of 201 patients) than patients who received other antibiotics (19.8%, *n* = 19 of 96 patients) or no antibiotic prophylaxis (17.9%, *n* = 12 of 67 patients). However, the differences in this subgroup analysis were not statistically significant.

We also analyzed whether the timing of antibiotic administration had an effect on the incidence of wound infection. For 230 patients from our cohort, we were able to track whether antibiotic administration occurred within 6 h before or after PEG placement (what was judged to be prophylaxis in line with guidelines). No significant difference, however, in the incidence of infections was found (*p* = 0.14) related to timing of antibiotic prophylaxis. Isolated pathogens in wound infections and antibiotic regimens upon PEG insertion are shown in Supplementary Table S3. A strikingly large number of yeast pathogens were isolated from swabs of wound infections (*n* = 121). Twenty-nine wound swabs showed *Candida* spp. (*Candida albicans* 53%, *Candida glabrata* 16.6%, *Candida krusei* 13.3%, Candida *tropicales* 3.3%, and other *Candida* spp. 13.3%) only (24%), with no bacterial coinfection. *Candida* infections were detected more frequently in patients receiving prophylactic antibiotics (57.9%, *n* = 33 of 57 patients) compared to patients not receiving antibiotic prophylaxis (20%, *n* = 3 of 15 patients), although the results were not statistically significant (*p* = 0.1).

## 4. Discussion

The incidence of wound infections after PEG tube insertion reported in the literature shows large variations from 4% to 30%. These marked differences are probably due to different study designs and heterogeneous patient populations, but they are also related, in particular, to differences in the length of the observation periods. In most studies, the observation period does not exceed the first 30 days after PEG tube insertion [11]. Hence, potential late complications, in particular wound infections, after PEG placement are not documented. The presented study used an observation period of 3 years in a large patient collective of 616 patients after PEG placement. We observed a relevant proportion of 25% wound infections in patients after PEG insertion and recurrent wound infections in 6.5% of the patients.

In this study, we identified several factors associated with an increased risk of post-interventional wound infections after PEG placement. Among them, a clear association between vascular risk factors and wound infections after PEG placement was shown, namely nicotine abuse and previous ischemic stroke. Smoking is a known factor that increases the risk of infection in general, by altering the structural and immunological defense mechanisms of the host [25]. Malignancies are considered risk factors for post-interventional wound infections after PEG placement [9]. We find a statistically significant association between head and neck cancer and wound infections, but no general association with other malignancies. In HNC patients we considered it likely that altered microbial colonization could have played a role due to spreading of bacteria from the tumor area to the point of entry of the PEG, analogous to the seeding of malignant cells in this tumor entity [26,27]. The increased incidence of tumor dissemination by the pull technique compared to the push technique reported in the literature shows that such cell dissemination is possible [10]. The results of the germ spectrum analyses in our study support this theory. We found *viridans streptococci* and *enterobacteria* that colonize the oropharyngeal tract in HNC patients more frequently in wound swabs from the PEG entry site in these patients [28]. However, infection rates were similar between pull and push cohorts in our study and no differences in pathogen patterns were observed between both insertion techniques, which argues against a role of bacterial spreading. These findings are consistent with the results of several other published studies [10].

In contrast to the technique of PEG placement, there seems to be a correlation between the oncological therapy concept and wound infections after PEG placement in malignant diseases. Simultaneous radio- and chemotherapy were associated with increased rates of wound infections in this study. Here, extensive mucositis is frequently observed, often plus a decrease in white blood cell counts, and possibly associated with microbial colonization and consecutive superinfections [29]. Both could contribute to the development of wound infections after PEG placement. There is only one study from India in patients with HNC that addresses pathogen colonization depending on the type of cancer treatment [14]. The authors of this study were able to demonstrate an increased prevalence of *Candida* spp. in wound swabs, which corresponded with the severity of the mucositis. Despite a high percentage of *Candida* spp. colonization of 30.1% in wound swabs, we did not detect *Candida* spp. disproportionately in HNC patients. Noteworthily, pathogen detection from PEG insertion site swabs cannot reliably distinguish between infection and colonization. To conclusively assess the role of an altered microbial spectrum and *Candida* colonization during radio-chemotherapy in the development of wound infections after PEG placement, further studies are needed.

To investigate the impact of antibiotic prophylaxis in all patients and of decolonization in MDRO-positive patients on wound infection rates after PEG placement, we investigated their occurrence within 7 days after PEG tube insertion. International guidelines recommend anti-infective prophylaxis with a third-generation cephalosporin 30 min before PEG placement [2,12,15]. In our study, only approximately half of the patients (52.3%) were treated according to the guideline recommendations. The reasons for apparent non-compliance with the guideline recommendations are ultimately unknown, however, are likely due to a lack of knowledge and a lack of awareness. About one fourth of the patients (23.1%) received no anti-infective prophylaxis at all. Interestingly, we did not see any difference in the number of wound infections in patients with or without guideline based anti-infective prophylaxis. However, there was a trend toward less severe wound infections and less need for anti-infective therapy in patients with guideline-based prophylaxis. Notably, studies in children have shown that anti-infective prophylaxis for PEG placement was not beneficial in this patient group [30]. A general benefit of anti-infective prophylaxis seems to be at least debatable and may need to be tailored to specific patient populations and local conditions, such as the local resistance situation. Eradication for MRSA prior to PEG tube placement is recommended by international guidelines if colonization is evident [2]. The rate of MRSA-positive patients in our cohort was only 1.6%, which was too low to draw meaningful conclusions. However, it is worth reporting that we diagnosed new MDROs after anti-infective prophylaxis in very few cases. Notably, anti-infective prophylaxis was given too inconsistently and often not according to the guidelines. Even if our data did not show a direct link to the appearance of MDROs, this use should at least be critically questioned and the use of anti-infectives in accordance with the guidelines should be worked towards.

In summary, our data demonstrate that infectious complications after PEG placement, especially wound infections, are frequent and play an important role in the clinic. Anti-infective prophylaxis in PEG placement, although not reducing the absolute incidence of wound infections, nevertheless minimizes the incidence of severe courses. Risk factors in our study were head and neck cancer and radio-chemotherapy as well as vascular pre-existing diseases. A more individualized approach to the patient’s particular risk profile and more appropriate use of anti-infective prophylaxis require further prospective studies. In the future, recommendations for peri-interventional anti-infective therapy and the frequency of follow-up of patients after PEG placement could be adapted according to the respective risk profile. However, the retrospective nature of our study does not currently allow a statement on the effectiveness of such strategies in reducing wound infections. Further multicenter analysis on the germ profiles of these patients could help to identify suitable strategies for prophylactic antibiotic administration, avoid unnecessary antibiotic administration, and thus reduce the development of antibiotic resistance.

## Figures and Tables

**Table 1:** Baseline characteristics and incidence of wound infections.

Characteristics	All Patients (*N* = 616)	No Wound Infection (*N* = 462)	Wound Infection (*N* = 154)	*p* Value
Age (years), median (range)	66 (19–95)	67 (10–95)	65 (14–91)	0.2
Male gender, *n* (%)	422 (68.5)	310 (67.1)	112 (72.7)	0.63
BMI < 17, *n* (%)	61 (9.9)	45 (9.7)	16 (10.4)	0.4
Nicotine, *n* (%)	107 (17.4)	70 (15.2)	37 (24.0)	**0.01**
Alcoholism, *n* (%)	63 (10.2)	37 (8.0)	26 (16.9)	0.27
Liver cirrhosis, *n* (%)	23 (3.7)	20 (4.3)	3 (1.9)	0.1
HIV, *n* (%)	15 (2.4)	14 (3.0)	1 (0.6)	0.13
Diabetes mellitus, *n* (%)	64 (10.4)	50 (10.8)	14 (9.1)	0.68
Cardiovascular disease, *n* (%)	195 (31.7)	139 (30.1)	56 (36.4)	0.1
Malignant disease, *n* (%)	424 (68.8)	288 (62.3)	136 (88.3)	**<0.001**
Head and neck cancer, *n* (%)	364 (59.1)	240 (51.9)	124 (80.5)	**<0.001**
Esophageal carcinoma, *n* (%)	22 (3.6)	17 (3.7)	5 (3.2)	0.82
Other cancer, *n* (%)	48 (7.8)	37 (8.0)	11 (7.1)	0.76
Ischemic Stroke, *n* (%)	53 (8.6)	14 (3.0)	39 (25.3)	**0.01**
Parkinson’s disease, *n* (%)	19 (3.1)	16 (3.5)	3 (1.9)	0.37
Dementia, *n* (%)	19 (3.1)	17 (3.7)	2 (1.3)	0.16
Amyotrophic lateral sclerosis, *n* (%)	12 (1.9)	10 (2.2)	2 (1.3)	0.51
Other neurological disease, *n* (%)	31 (5.0)	24 (5.2)	7 (4.5)	0.02
Malnutrition, *n* (%)	7 (1.1)	6 (1.3)	1 (0.6)	0.52

**Table 2:** Complications overall and complications in patients with and without head and neck cancer.

Complications	All Patients (*N* = 616)	Head and Neck Cancer (*N* = 364)	Others (*N* = 252)	*p* Value
No complications, *n* (%)	427 (69.3)	224 (61.5)	203 (80.6)	**<0.001**
Wound infection, *n* (%)	154 (25)	124 (34.1)	30 (12.9)	**<0.001**
Buried bumper, *n* (%)	7 (1.1)	7 (1.9)	0 (0)	**0.027**
Aspiration, *n* (%)	5 (0.8)	0 (0)	5 (2)	**0.007**
Leakage, *n* (%)	3 (0.5)	1 (0.3)	2 (0.8)	0.36
Dislocation, *n* (%)	13 (2.1)	8 (2.2)	5 (2)	0.86
Removal for other reasons, *n* (%)	2 (0.3)	1 (0.3)	1 (0.4)	0.79

**Table 3:** Multivariate regression analysis of wound infections within seven days after PEG placement.

Secondary Diagnosis	OR (95% CI)	*p* Value
Age > 70 years	0.7 (0.39–1.24)	0.31
BMI < 17	0.95 (0.61–1.49)	0.83
Nicotine	0.83 (0.5–1.38)	0.48
Alcoholism	0.7 (0.39–1.24)	0.22
Liver cirrhosis	0.49 (0.2–1.17)	0.1
HIV	4.7 (0.61–36.14)	0.14
Diabetes mellitus	1.14 (0.61–2.13)	0.69
Cardiovascular disease	0.8 (0.53–1.2)	0.29
Head and neck cancer	0.23 (0.05–0.95)	**0.04**
Esophageal carcinoma	0.47 (0.09–2.23)	0.34
Ischemic stroke	1.41 (0.48–4.17)	0.53
Parkinson’s disease	0.49 (0.13–2.7)	0.49
Dementia	0.85 (0.22–6.24)	0.85
Amyotrophic lateral sclerosis	0.4 (0.06–2.6)	0.34

**Table 4:** Treatment during and after PEG placement of patients with or without wound infection.

	All Patients (*N* = 616)	No Wound Infection (*N* = 462)	Wound Infection (*N* = 154)	*p* Value
Radio-chemotherapy. *n* (%)	325 (52.8)	216 (66.5)	109 (33.5)	**<0.001**
Radiation. *n* (%)	27 (4.4)	21 (77.8)	6 (22.2)	0.45
Surgery. *n* (%)	64 (10.4)	52 (81.3)	12 (18.8)	0.22

**Table 5:** Detected germ spectrum in wound swaps after PEG placement (*N* = 224).

Pathogen	Total (*N* = 402)	Head and Neck Cancer (*N* = 337)	Other (*N* = 67)	*p* Value
*Staphylococcus aureus*, *n* (%)	40 (9.9)	36 (10.7)	4 (5.8)	0.24
*Coagulase-negative staphylococci*, *n* (%)	29 (7.2)	26 (7.7)	5 (7.5)	0.90
*Viridans streptococci*, *n* (%)	21 (5.2)	21 (6.2)	0 (0)	**0.036**
*Enterococci*, *n* (%)	27 (6.7)	21 (6.2)	6 (8.9)	0.41
*Enterococcus faecalis*, *n* (%)	16 (3.9)	13 (3.9)	3 (4.5)	0.81
*Enterococcus faecium*, *n* (%)	11 (2.7)	8 (2.4)	3 (4.5)	0.33
*Enterobacterales*, *n* (%)	72 (17.9)	57 (16.9)	15 (22.4)	0.28
*Enterobacter cloacae*, *n* (%)	25 (6.2)	21 (6.2)	4 (5.9)	0.94
*Escherichia coli*, *n* (%)	14 (3.5)	11 (3.3)	3 (4.5)	0.62
*Proteus* spp. (*Proteus mirabilis*, *Proteus penneri*, *Proteus vulgaris*), *n* (%)	9 (2.9)	7 (2.1)	2 (2.9)	0.65
*Serratia marcescens*, *n* (%)	4 (0.9)	4 (1.3)	0 (0)	0.37
*Citrobacter freundii*, *n* (%)	1 (0.3)	0	1 (1.5)	0.02
*Enterobacter aerogenes*, *n* (%)	1 (0.3)	1 (0.3)	0 (0)	0.66
*Klebsiella* spp. (*Klebsiella pneumoniae*, *Klebsiella oxytoca*), *n* (%)	18 (6.4)	13 (3.9)	5 (7.5)	0.19
*Haemophilus* spp. *n* (%)	5 (1.8)	5 (1.5)	0 (0)	0.31
*Haemophilus parainfluenzae*, *n* (%)	2 (0.5)	2 (0.6)	0 (0)	0.53
*Haemophilus influenzae*, *n* (%)	3 (0.7)	3 (0.9)	0 (0)	0.44
*Pseudomonas aeruginosa*, *n* (%)	32 (11.4)	25 (7.4)	7 (17.1)	0.4
Normal upper respiratory tract flora, *n* (%)	4 (1.4)	4 (1.2)	0 (0)	0.37
Normal skin flora, *n* (%)	51 (18.2)	41 (12.2)	10 (14.9)	0.53
Total of bacterial pathogens, *n*	281	242	41	0.083
*Candida* spp. *n* (%)	121 (30.1)	95 (28.2)	26 (38.8)	0.083
*Candida albicans*, *n* (%)	72 (17.9)	59 (17.5)	13 (19.4)	0.71
*Candida* spp. non albicans *n* (%)	49 (12.2)	36 (10.7)	13 (19.4)	0.05

**Table 6:** Type of wound infection after PEG placement.

Type of Wound Infection	With Antibiotic Prophylaxis (*N* = 474)	Without Antibiotic Prophylaxis (*N* = 142)	*p* Value
Minor peristomal infection, *n* (%)	9 (1.9)	3 (2.1)	0.87
Need of systemic antibiotic treatment, *n* (%)	34 (7.1)	16 (11.3)	**0.1**
Sepsis, *n* (%)	1 (0.2)	0 (0)	0.58
Mycotic infection, *n* (%)	8 (1.7)	1 (0.7)	0.37
**Total, *n* (%)**	52 (11)	20 (14.1)	0.31

**Table 7:** Most commonly used antibiotic classes and incidence of wound infection after univariate and multivariate regression analysis.

Antibiotics	Total (*N* = 474)	Wound Infection (*N* = 72)	*p* Value (Univariate)	*p* Value (Multivariate)
Ceftriaxone (single shot), *n* (%)	248 (52.3)	30 (12.0)	0.79	0.68
Ceftriaxone (long term), *n* (%)	25 (5.3)	3 (4.2)	0.96	0.95
Other cephalosporine, *n* (%)	41 (8.6)	7 (9.7)	0.27	0.47
Fluoroquinolone, *n* (%)	57 (12.0)	3 (4.2)	0.12	0.12
Penicillin, *n* (%)	52 (10.9)	7 (9.7)	0.67	0.69
Macrolide, *n* (%)	18 (3.8)	1 (1.4)	0.42	0.41
Carbapenem, *n* (%)	64 (13.5)	4 (5.6)	0.16	0.31
Glycopeptide, *n* (%)	30 (6.3)	2 (2.7)	0.39	0.66
Linezolid, *n* (%)	7 (1.5)	0 (0.0)	0.93	0.97

## Data Availability

The data presented in this study are available on request from the corresponding author. The data are not publicly available due to privacy restrictions.

## Supplementary Materials

The following supporting information can be downloaded at: https://www.mdpi.com/article/10.3390/jcm12093175/s1, Table S1: Subgroup analysis of patients with head and neck cancer; Table S2: Pre-existing and newly diagnosed MDR bacteria after PEG placement; Table S3: Spectrum of the most common pathogens in relation to the antibiotic prophylaxis used; Table S4: Heat map of resistance analysis of the most frequently isolated s pathogens (natural and acquired resistance are indicated in percent).
