# Supplementary material for: Risk Factors and Role of Antibiotic Prophylaxis for Wound Infections after Percutaneous Endoscopic Gastrostomy
Source: J Clin Med. 2023 Apr 28;12(9):3175. doi: 10.3390/jcm12093175 (PMC10179185; doi:10.3390/jcm12093175)
Supplement: Supplementary file 1 [file jcm-12-03175-s001.zip › jcm-2219509-supplementary.pdf]

SUPPLEMENT

Table S1. Subgroup analysis of patients with head and neck cancer.

**Patients with head and neck cancer (N=364)**			
**Secondary diagnosis**	**Total (N=364)**	**Wound infection (N=124)**	***P* value**
Age > 70 years, (n, %)	112, 30.8	12, 10.7	0.47
BMI < 17 kg/m², (n, %)	32, 8.8	9, 28.1	0.3
Nicotine, (n, %)	273, 75	41, 15.0	0.34
Alcoholism, (n, %)	305, 83.8	50, 16.4	0.23
Cardiovascular disease, (n, %)	131, 35.9	39, 29.8	0.74
Liver cirrhosis, (n, %)	17, 4.7	9, 53.9	**0.03**
HIV infection, (n, %)	3, 0.8	0, 0	0.9
Diabetes mellitus, (n, %)	21, 5.7	12, 57.1	0.34
Cerebrovascular disease, (n, %)	10, 2.7	3, 30	0.76
Dementia, (n, %)	3, 0.8	2, 66.7	0.39
Amyotrophic lateral sclerosis, (n, %)	0	0, 0	
Parkinson's disease, (n, %)	2, 0.5	1, 50	**0.04**
Other cerebral diseases, (n, %)	1, 0.3	0, 0	0.94

Table S2. Pre-existing and newly diagnosed MDR bacteria after PEG placement.

**MDR bacteria**	**Pre-existing colonization All patients (N=616)**	**Pre-existing colonization HNC patients (n=364)**	**Newly diagnosed in wound swap of PEG infection**
MRSA, n (%)	10 (1.6)	5 (1.4)	0
VRE, n (%)	25 (4.0)	4 (1.1)	0
3 MRGN, n (%)	31 (5.0)	10 (2.7)	2*
4 MRGN, n (%)	16 (2.6)	5 (1.4)	1**

*Both *E. coli* resistant to Piperacillin/Tazobactam, third generation Cephalosporines and Fluoroquinolones
** *Klebsiella pneumoniae* resistant to the above mentioned and Carbapenems

Table S3 Spectrum of the most common pathogens in relation to the antibiotic prophylaxis used.

		**Gram-positive bacteria**				**Gram-negative bacteria**						
**Antibiotics**	**Total**	**Staphylococcus aureus**	**Coagulase-negative staphylococci**	**Viridans streptococci**	**Enterococci**	**Enterobacterales**	**Haemophilus**	**Klebsiella**	**Pseudomonas aeruginosa**	**Upper respiratory tract**	**Normal skin flora**	**Candida**
No antibiotics, n (%)	20 (14.1)	2 (10)	4 (20)	1 (5)	2 (10)	3 (15)	0	1 (5)	3 (15)	1 (5)	0	1 (5)
Aminopenicillin, n (%)	1 (10)	0	0	0	0	1 (100)	0	0	0	0	0	0
Piperacillin/Tazobactam, n (%)	1 (2.7)	0	0	0	1 (100)	0	0	0	0	0	0	1 (100)
Cephalosporine 2nd, n (%)	6 (20)	1 (17)	0	0	1 (17)	3 (50)	0	1 (17)	0	0	4 (67)	4 (67)
Cephalosporine 3rd, n (%)	29 (11.2)	4 (14)	5 (17)	4 (14)	2 (7)	0	0	0	1 (3)	1 (3)	3 (10)	16 (55)
Carbapenems, n (%)	4 (6.3)	0	0	0	2 (50)	0	1 (25)	0	0	0	0	3 (75)
Fluoroquinolone 2nd, n (%)	1 (3.7)	0	0	0	0	0	0	0	1 (100)	0	0	1 (100)
Fluoroquinolone 3rd, n (%)	1 (4.8)	0	0	0	0	0	0	1 (100)	1 (100)	0	0	1 (100)

Table S4 Heat map of resistance analysis of the most frequently isolated s pathogens (natural and acquired resistance are indicated in percent).

	Gram-positive bacteria				Gram-negative bacteria				
**Resistance**	**Staphylococcus aureus (N=7)**	**Coagulase-negative staphylococci (N=9)**	**Viridans streptococci (N=5)**	**Enterococci (N=8)**	**Enterobacterales (N=7)**	**Haemophilus (N=1)**	**Klebsiella (N=3)**	**Pseudomonas (N=6)**	**Candida (N=45)**
Penicillin G, %	100	100	0	0	100	100	100	100	
Ampicillin, %	100	100	0	13	86	0	100	100	
Amoxicillin/Clavulanic acid, %	0	11	0	13	86	0	33	100	
Piperacillin/Tazobactam, %	0	0	0	0	57	0	0	0	
Cephalosporine 2nd, %	0	67	0	100	0	0	33	100	
Cephalosporine 3rd, %	0	67	0	100	86	0	33	100	
Cefotaxime, %	0	0	0	100	57	0	33	100	
Ceftriaxone, %	0	0	0	100	0	0	0	100	
Carbapenem, %	0	11	0	25	0	0	0	17	
Fluoroquinolone 2nd, %	0	0	100	100	14	0	33	0	
Fluoroquinolone 3rd, %	0	33	0	50	0	0	33	0	
Macrolide, %	100	100	60	100	100	0	100	100	
Glycopeptide, %	0	0	0	0	100	100	100	100	
Fluconazole, %									4
